# Analysis of the role of IL-1 family and related genes in head and neck squamous cell carcinoma

**DOI:** 10.1016/j.bjorl.2024.101484

**Published:** 2024-09-11

**Authors:** Gaofei Yin, Wei Guo, Rong Wang, Nuan Li, Xiaohong Chen, Yang Zhang, Zhigang Huang

**Affiliations:** Beijing Tongren Hospital, Capital Medical University, Beijing, China

**Keywords:** IL-1, Head and neck squamous cell carcinoma, Databases, SIGIRR

## Abstract

**Objective:**

The Interleukin-1 (IL-1) family plays an important regulatory role in the development of tumors, but its function is still unclear in head and neck squamous cell carcinoma (HNSCC). Analyzing the IL-1 family can help to understand the tumor mechanism.

**Methods:**

Using GEPIA2, UALCAN, cBioprotocol and HPA databases, the IL-1 family and related genes (IL-1α, IL-1β, IL1RN, IL1R1, IL1R2, IL1RL1, IL1RL2, IL1RAP, IL1RAPL1, IL1RAPL2, IL1F10, IL18, IL18BP, IL18R1, IL18RAP, IL36A, IL36B, IL36G, IL36RN, IL33, IL37, SIGIRR, CASP1, AIM2) were analyzed for their expression and prognostic relevance in HNSCC. The Kaplan-Meier, log-rank test and Spearman correlation were used to analysis.

**Results:**

In tumors, IL-1α, IL-1β, IL1R1, IL1RL1, IL1F10, IL33, CASP1, and AIM2 are highly expressed, while IL1RN, IL1RAPL1, IL1RAPL2, IL18BP, IL18R1 and IL18RAP are poorly expressed. IL-1α, IL1RAP, and IL1RAPL2 were prognostic risk factors in at least two databases, while IL18RAP, IL36A, and SIGIRR were prognostic protective factors. SIGIRR was confirmed in all three databases. Compared to HPV- tumors, IL18RAP and SIGIRR are highly expressed in HPV+ tumors. In addition, IL-1α, IL-1β, IL1RL2, IL1RAP were negatively correlated with CD8A/B expression, while IL1R2, IL18R1, IL18RAP, IL33, SIGIRR, CASP1, AIM2 were positively correlated with CD8A/B expression.

**Conclusion:**

The differential expression of the IL-1 family and related genes affects the microenvironment changes and survival prognosis of HNSCC patients. Among them, IL-1α, IL1RAP, IL18RAP, and SIGIRR may affect the prognosis of patients by affecting local CD8+ T cell infiltration in the tumor.

**Level of evidence:**

3.

## Highlights

• Using databases to analyze IL-1 and related genes for their relevance in HNSCC.
• We found IL18RAP and SIGIRR are highly expressed in HPV+ tumors.
• IL18RAP, IL36A and SIGIRR were found to be prognostic protective factors.
• IL-1A, IL1RAP, IL18RAP and SIGIRR affect the prognosis by CD8+ T-cell infiltration.

## Introduction

The Interleukin-1 (IL-1) family consists of several members: IL-1α、IL-1β、 IL-1Ra, IL-18, IL-33, IL-36α、IL-36β、IL-36γ、IL-36Ra, IL-37 and IL-38. All members have similar gene structures, C-terminal amino acid homology, and conserved tertiary protein conformations.^1^ Moreover, these cytokines have intracellular domains homologous to Toll-like Receptors (TLRs) and share a common signaling pathway with TLRs, thereby placing them at the apex of immune responses against various exogenous and endogenous danger signals.^2^ Therefore, dysregulation of IL-1 cytokine activity often leads to immune-related effects, mediating autoimmune inflammatory diseases and tumor development.^3^ IL-1 family members also have complex and diverse roles in controlling carcinogenesis and tumor progression. Notably, added evidence^4^, ^5^ suggests that the development of Head and Neck Squamous Cell Carcinoma (HNSCC) is associated with chronic inflammation, and IL-1 is involved as a strong pro-inflammatory cytokine commonly found at the tumor site. Our previous research has also confirmed this point.^6^ However, the specific role of the IL-1 family in HNSCC is unclear. The regulatory mechanism of the IL-1 family in HNSCC seems more complex. Thus, this article analyzes the data of HNSCC patients in existing databases to explore the specific roles of the IL-1 family and related genes in HNSCC.

## Methods

### IL-1 family and related genes

This is a retrospective analysis based on database data. The genes involved in the article include members of the IL-1 family and related genes. IL-1 family members: IL-1α, IL-1β, IL1F10, IL18, IL36A, IL36B, IL36G, IL33, IL37; IL-1 related receptors and receptors: IL1R1, IL18R1, IL1RL1, IL1RL2, IL1RAP, IL1RAPL1, IL18RAP; Negative regulatory factors: IL1R2, IL1RN, IL36RN, IL18BP, SIGIRR; Inflammatory bodies: CASP1 and AIM2.Then we searched for the correlation between IL-1 related genes and HNSCC using public databases in January 2024.

### GEPIA2 database

The GEPIA2 database (http://gepia2.cancer-pku.cn/#index) is an updated version of GEPIA. It can be used to analyze the RNA expression sequencing data of 9736 tumor samples and 8587 normal samples in TCGA and GTEx. We used this database to analyze the expression of the IL-1 family and related genes in 519 HNSCC patients.

### UALCAN database

The UALCAN database (http://ualcan.path.uab/index.hrml) is a comprehensive, interactive Web resource. In the TCGA module, after the input of the target genes IL-1α, IL-1β, IL1RN, IL1R1, IL1R2, IL1RL1, IL1RL2, IL1RAP, IL1RAPL1, IL1RAPL2, IL1F10, IL18, IL18BP, IL18R1, IL18RAP, IL36A, IL36B, IL36G, IL36RN, IL33, IL37, SIGIRR, CASP1, and AIM2 the expression of genes between normal tissue and cancer was analyzed online (564 patients). Subsequently, we analyzed the correlation between the IL-1 family and related genes and HPV infection status in 168 patients.Further analysis was conducted on the Overall Survival (OS) rate of patients with different gene expression levels.

### HPA database

The HPA database (https://www.proteinatlas.org) is based on proteomics, transcriptomics, and systems biology. As such, it provides information on the tissue and cellular distribution of 24,000 human proteins. The protein expression data and clinical information of normal and tumor tissues were also included. We analyzed the OS of different protein expressions in 499 patients.

### cBioprotal database

The cBioprotal database (http://www.cbioportal.org/) contains data from databases such as TCGA, ICGC, GEO, etc. The integrated genomic data types include somatic mutations, DNA Copy Number Changes (CNAs), mRNA and miRNA expression levels, DNA methylation, protein abundance, and phosphoprotein abundance. Using this database, we analyzed the correlation between the IL-1 family and related gene expression of CD8A/B in 520 patients.

### Statistical analysis

Correlation datasets for the differential expression of cancer and normal tissues were created in UALCAN with *p*-values, which were also listed as median values. Survival curves were drawn using GEPIA2, UALCAN through the Kaplan-Meier curve. The statistical difference between the curves were measured by the log-rank test. The *p*-values were used to compare OS among patients in different groups using the GEPIA2, UALCAN, and HPA. The correlation of gene expression was analyzed in cBioprotal, where Spearman’s correlation was employed as the correlation coefficient. A *p-*value <0.05 was examined to be statistically significant throughout the text. Note that the data comes from TCGA and other websites.

## Results

### Differences in the expression of IL-1 family and related genes between normal and tumor tissues

**Figure 1:**
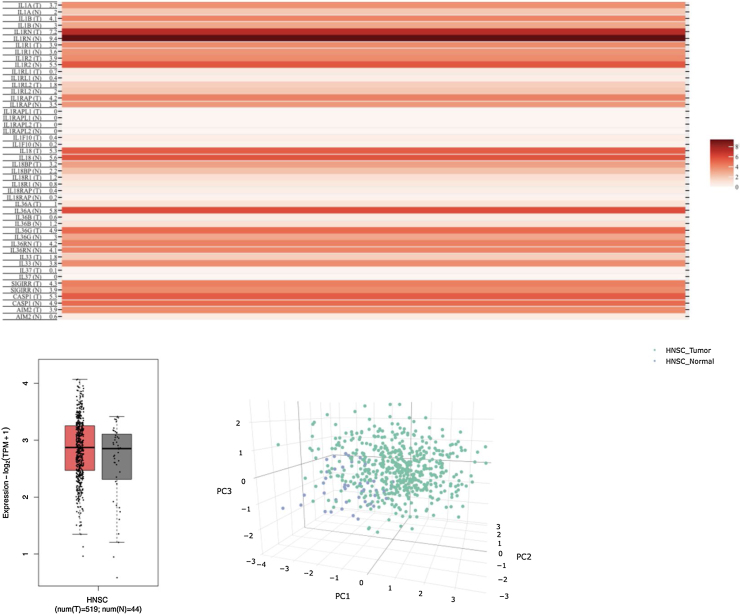
(A) Gene comparisons using a matrix plot in Multiple gene comparison; (B) Gene expression comparisons in HNSCC; (C) PCA of multiple genes and cancer types.

Using the GEPIA2 database to analyze the differential expression of the IL-1 family and related genes in HNSCC and normal tissues (Fig. 1A), it was found that the IL-1 family and related mRNA showed relatively high expression in tumor tissues (Fig. 1B). Furthermore, Principal Component Analysis (PCA) of multiple genes and HNSCC in PCA reveals significant distribution differences between normal and tumor tissues (Fig. 1C).

**Figure 2:**
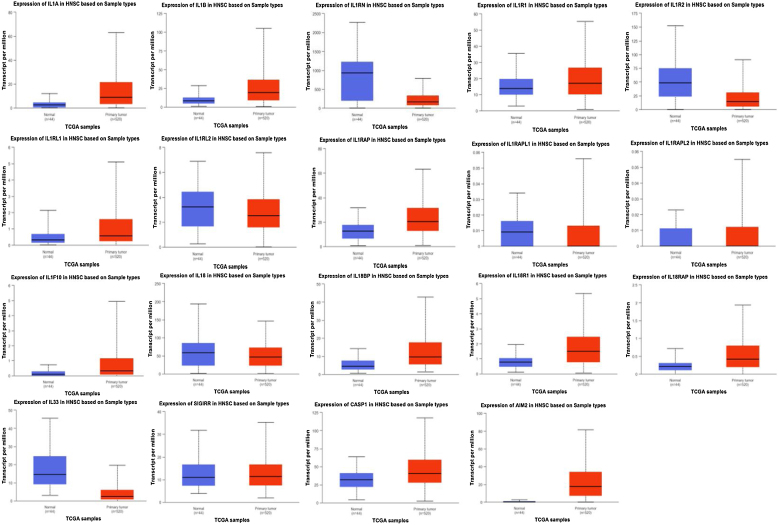
Expression differences of the IL-1 family and related genes between normal and tumor tissues in the UALCAN database.

The UALCAN database analysis of the specific differential analysis and *p-*values of the IL-1 family and related gene expression in normal and tumor tissues are shown in Table 1. In tumor tissues, IL-1α, IL-1β, IL1R1, IL1RL1, IL1F10, IL33, CASP1, and AIM2 are highly expressed. The difference in high expression of IL-1α, IL-1β, IL1R1, IL1RL1, IL1F10, IL33, IL1R2, IL18, CASP1 and AIM2 is statistically significant (*p* < 0.01). Moreover, IL1RAPL1, IL1RAPL2, IL18BP, IL18R1, IL18RAP, and SIGIRR were low in expression, and the expression differences of IL1RN, IL1RAPL1, IL18BP, IL18R1, and IL18RAP were statistically significant (*p* < 0.01) (Fig. 2).

**Table 1:** Analysis of the differential expression of the IL-1 family and related genes in normal and tumor tissues.

	Normal (44) (Median)	Tumor (520) (Median)	*p*
IL-1α	2.214	8.824	<0.01
IL-1β	8.475	19.131	<0.01
IL1RN	931.233	164.013	<0.01
IL1R1	13.786	17.11	<0.01
IL1R2	48.829	14.122	>0.05
IL1RL1	0.318	0.575	<0.01
IL1RL2	3.237	2.53	>0.05
IL1RAP	12.634	20.385	<0.01
IL1RAPL1	0.009	0	<0.01
IL1RAPL2	0	0	>0.05
IL1F10	0.097	0.329	<0.01
IL18	58.061	46.445	>0.05
IL18BP	4.408	9.658	<0.01
IL18R1	0.77	1.491	<0.01
IL18RAP	0.21	0.42	<0.01
IL33	14.496	2.341	<0.01
SIGIRR	10.928	11.324	>0.05
CASP1	31.837	40.729	<0.01
AIM2	0.415	17.515	<0.01

**Figure 3:**
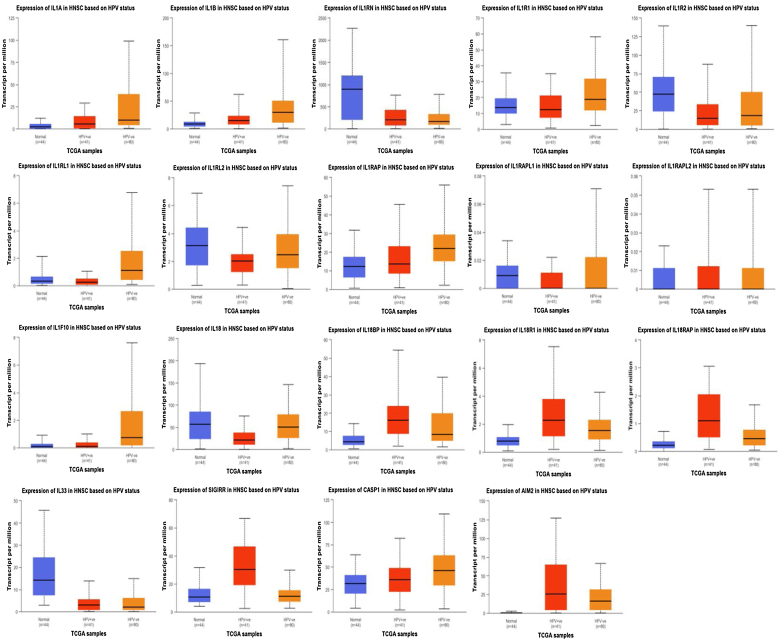
Differential expression and analysis of the IL-1 family and related genes in normal tissues and HPV- and HPV+ tumor tissues in the UALCAN database.

Further analysis of the differential expression of IL-1 family and related genes in normal tissues and HPV- and HPV+ tumor tissues (Table 2) revealed that IL-1α, IL-1β, IL1R1, IL1R2, IL1RL1, IL1RAP, IL1F10, IL-18 and CASP1 were highly expressed in HPV- tumors. Among these, the expression of IL-1α, IL-1β, IL1R1, IL1R10, and IL1RAP was significantly higher than that of HPV+ and normal tissues, and the difference was statistically significant. Additionally, IL18 expression is higher in HPV tumors than in HPV+ tumors, and the difference is statistically significant. However, overall IL18 expression is lower than in normal tissues. IL1RN, IL18BP, IL18R1, IL18RAP, SIGIRR, and AIM2 are highly expressed in HPV+ tumors, with significantly higher levels of IL18BP, IL18R1, IL18RAP, and AIM2 compared to HPV- and normal tissues. Compared to normal tissues and HPV tumors, SIGIRR is highly expressed in HPV+ tumors, and the difference is statistically significant. Although IL1RN expression is higher in HPV+ tumors than in HPV- tumors, overall IL1RN expression is lower than in normal tissues (Fig. 3).

**Table 2:** Differential analysis of the IL-1 family and related gene expression in normal tissues and HPV- and HPV+ tumor tissues.

	A: Normal (44) (Median)	B: Tumor HPV+ (41) (Median)	C: Tumor HPV- (80) (Median)	*p*
IL-1α	2.214	5.588	9.822	<0.01 (A:B:C)
IL-1β	8.94	15.251	29.719	<0.01 (A:B:C)
IL1RN	892.49	208.496	169.697	<0.01 (A:C)
IL1R1	13.629	12.44	18.732	<0.01 (A:C/B:C)
IL1R2	47.174	14.815	18.249	>0.05
IL1RL1	0.323	0.262	1.112	<0.01 (A:C/B:C)
IL1RL2	3.127	2.019	2.478	>0.05
IL1RAP	12.261	13.548	21.875	<0.01 (A:B:C)
IL1RAPL1	0.009	0	0	>0.05
IL1RAPL2	0	0	0	>0.05
IL1F10	0.11	0.1	0.743	<0.01 (A:C/B:C)
IL-18	56.508	21.354	50.384	<0.01 (A:C/B:C)
IL18BP	4.344	16.149	8.278	<0.01 (A:B/A:C)
IL18R1	0.781	2.271	1.547	<0.01 (A:B:C)
IL18RAP	0.217	1.101	0.453	<0.01 (A:B:C)
IL33	14.211	3.061	2.102	<0.01 (A:B/A:C)
SIGIRR	10.629	30.326	11.138	<0.01 (A:B/B:C)
CASP1	31.629	35.796	46.118	<0.01 (A:B/B:C)
AIM2	0.403	25.505	15.873	<0.01 (A:B:C)

### The correlation between the expression of the IL-1 family and related genes and the prognosis of HNSCC

We analyzed the impact of the IL-1 family and related gene expression levels on overall patient survival through GEPIA2, UALCAN, and HPA databases. In the GEPIA2 database, it can be found that the expression levels of IL1RAPL2, IL18RAP, IL36A, and SIGIRR significantly impact the overall survival of patients. High expression of IL1RAPL2 is a risk factor affecting patient survival, while high expression of IL18RAP, IL36A, and SIGIRR is a protective factor affecting patient survival. In the UALCAN database, we found that IL-1A, IL1RAP, and SIGIRR expression levels significantly impact overall patient survival. Additionally, high expression of IL-1A and IL1RAP is a risk factor affecting patient survival, while high expression of SIGIRR is a protective factor affecting patient survival. In the HPA database, the expression levels of IL-1A, IL1RN, IL1RL1, IL1RAP, IL1RAPL2, IL18RAP, IL36A, IL36B, IL36RN, IL37 and SIGIRR are correlated with overall survival prognosis (Table 3). Furthermore, high expression of IL-1A, IL1RL1, IL1RAP, and IL1RAPL2 is a risk factor affecting patient survival, while high expression of IL1RN, IL18RAP, IL36A, IL36B, IL36RN, IL37, and SIGIRR is a protective factor affecting patient survival. Notably, the three databases are consistent in the analysis of samples.

**Table 3:** Correlation analysis of the IL-1 family and related gene expression levels with 5-year overall survival in HNSCC tissues of HPA database.

499 patients	5-year survival (high)	5-year survival (low)	*p*
IL-1α	48%	47%	0.008
IL-1β	45%	47%	0.071
IL1RN	49%	36%	0.036
IL1R1	49%	34%	0.071
IL1R2	49%	44%	0.14
IL1RL1	35%	49%	0.020
IL1RL2	43%	49%	0.26
IL1RAP	31%	49%	0.0088
IL1RAPL1	45%	46%	0.40
IL1RAPL2	39%	59%	0.00015
IL1F10	58%	42%	0.063
IL18	45%	48%	0.16
IL18BP	54%	41%	0.078
IL18R1	51%	42%	0.071
IL18RAP	50%	40%	0.012
IL36A	48%	36%	0.0097
IL36B	58%	40%	0.036
IL36G	52%	40%	0.19
IL36RN	57%	40%	0.0087
IL33	47%	45%	0.16
IL37	52%	40%	0.026
SIGIRR	53%	42%	0.00091
CASP1	44%	48%	0.12
AIM2	50%	43%	0.34

**Figure 4:**
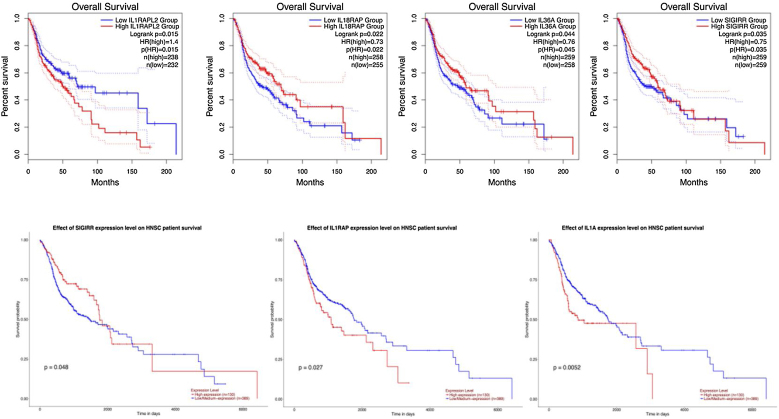
Correlation analysis between the IL-1 family and related gene expression levels and overall survival in tumor patients in GEPIA 2 and UALCAN databases.

**Figure 5:**
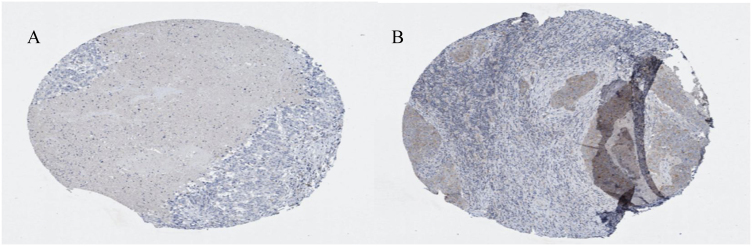
Immunohistochemical expression of SIGIRR in normal and tumor tissues of HNSCC using the HPA database. (A) Normal issue; (B) Tumor issue.

Based on the analysis of the results from three databases, IL-1A, IL1RAP, IL1RAPL2, IL18RAP, IL36A, and SIGIRR were associated with prognosis in at least two databases (Fig. 4). Among them, IL-1A, IL1RAP, and IL1RAPL2 were prognostic risk factors, while IL18RAP, IL36A, and SIGIRR were prognostic protective factors. Moreover, SIGIRR was confirmed to be a prognostic protective factor in all three databases (Fig. 5).

### The correlation between the expression of IL-1 family and related genes and CD8A/B

**Figure 6:**
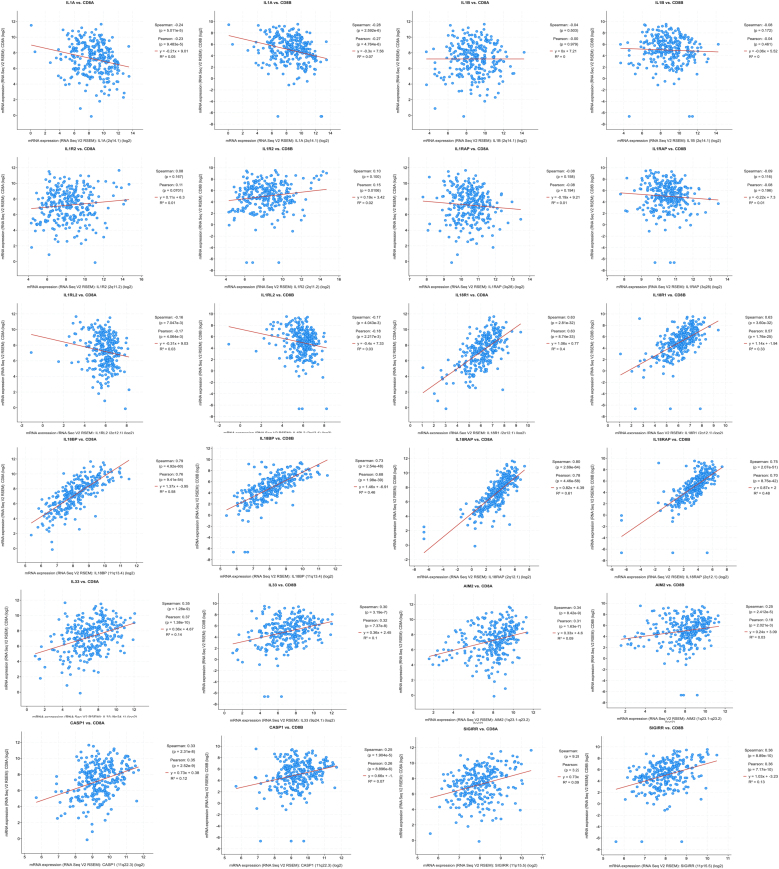
Correlation analysis of IL-1 and its family genes with CD8+ T-cells, CD8A, and CD8B expression in the cBioprotal database.

cBioprotal is used to analyze the correlation between IL-1 and its family genes and the expression of CD8+ T-cells, CD8A, and CD8B. As such, we analyzed the TCGA data queue, which had a total of 520 patients and 522 sample tissue information. Through correlation analysis, it can be found that the expression of IL-1α, IL-1β, IL1R2, IL1RAP, IL18BP, IL18R1, IL18RAP, IL33, SIGIRR, CASP1 and AIM2 is correlated with the expression of CD8A and CD8B. Among these, the expression of IL-1α, IL-1β, IL1RL2, and IL1RAP is negatively correlated with the expression of CD8A and CD8B, while the expression of IL1R2, IL18BP, IL18R1, IL18RAP, IL33, SIGIRR, CASP1, and AIM2 is positively correlated with the expression of CD8A and CD8B (Fig. 6).

## Discussion

The IL-1 family is a group of heterogeneous proteins that regulate various biological functions, including immunity. The 12 cytokines contained in the IL-1 family can be classified into receptor agonists (IL-1) based on their different functions: IL-1α, IL-1 β, IL-18, IL-33, IL-36 α, IL-36 β, IL-36 γ, and IL-37. Receptor antagonists (IL-1Ra, IL-36Ra, IL-38) are cytokines that emit signals through interactions with their corresponding receptors and co-receptors. In addition, receptor antagonists includes a group of IL-1 family orphan receptors, including a single Ig IL-1 related receptor (SIGIRR, also known as TIR8), IL-1RAPL1 (TIGIR-2), and IL-1RAPL2 (TIGIR-1).^7^ IL-1-mediated tumor promotion has been confirmed in different mouse and human tumors, including sarcoma, melanoma, pancreatic ductal adenocarcinoma, and breast cancer.^8^ Moreover, added evidence suggests that the development of head and neck squamous cell carcinoma is associated with chronic inflammation,^9^ and the role of IL-1 autocrine and paracrine signaling in TME is at the core of HNSCC development. This signal axis not only leads to an increase in the expression of proteases and factors but also assists in tumor cell invasion and metastasis.^10^, ^11^ Our center's previous research on the IL-1 family has involved IL-1α And IL-1β. It has been confirmed that both IL-1α And IL-1β have a tumor regulatory role in HNSCC.^6^, ^12^ However, further validation is needed regarding the role of other cytokines in the IL-1 family in HNSCC. In this study, it was found that IL-1α, IL-1β, IL1R1, IL1RL1, IL1F10, IL33, CASP1, and AIM2 were highly expressed in head and neck tumors, while IL1RN, IL1RAPL1, IL1RAPL2, IL18BP, IL18R1, and IL18RAP were low in expressed, which is consistent with previous literature. Based on the prognosis and expression correlation analysis with CD8+ T-cells, we also found that IL-1A and IL-1RAP are prognostic risk factors and negatively correlated with CD8A/B expression. At the same time, IL-18RAP and SIGIRR are prognostic protective factors and positively correlated with CD8A/B expression. This further confirms that the prognosis of HNSCC is related to the expression of local CD8+ T-cells in the tumor, and compared to HPV tumors, IL-18RAP and SIGIRR are highly expressed in HPV+ tumors. Therefore, we further analyzed the roles of IL-1A, IL1RAP, IL18RAP, and SIGIRR in previous literature and tumors.

The role of IL-1A in the occurrence and development of head and neck tumors is complex. It can promote tumor cell proliferation and enhance its anti-apoptotic ability while inducing angiogenesis, providing nutrients and oxygen to the tumor, thereby promoting its growth and diffusion. In addition, IL-1A can regulate the function and activity of immune cells, affecting the immune response of head and neck tumors.^13^ In Leon et al.'s study,^14^ it was confirmed that high expression of IL-1 is associated with the metastasis of HNSCC. The risk of metastasis is 5.3 times higher in patients with elevated levels, and the five-year survival rate without distant metastasis is reduced by 25%. Moreover, the expression of MMP-9 (a Matrix Metalloproteinase associated with EMT), PGE2 (a product activated by COX-2 associated with OSCC metastasis), VEGF (the most important angiogenic factor in HNSCC), and CXCL8 are also associated with different genes associated with metastasis, especially MMP-9 (a matrix metalloproteinase associated with EMT). IL-1A may also promote carcinogenesis under necrotic conditions. Notably, IL-1A is an effective activator of anti-tumor cytotoxic lymphocytes (such as NK cells and CD8+ cells), and IL-1A derived from necrotic cells may mainly serve as a warning for promoting carcinogenic inflammation.^15^

IL-1RAP, as a member of the IL-1 receptor family, was first identified as a co-receptor of type I and type II IL-1 receptors (IL-1RI and IL-1RII) in 1995.^16^ IL-1RAP is important in many stages of tumor promotion, progression, and metastasis. IL-1RAP is also overexpressed in various cancers, and studies have shown that downregulating IL-1RAP leads to a decrease in primary cell colony formation ability and an increase in apoptosis. IL-1RAP also plays a vital role in other solid and hematologic cancers. For example, overexpression (81%) is present in pancreatic ductal adenocarcinoma and gastric adenocarcinoma,^17^, ^18^ and inhibition of IL-1RAP reduces the survival ability and colony growth ability of tumor cells, leading to decreased invasiveness. Compared with normal tissue, the expression of IL-1RAP in cervical cancer tissue has also increased. IL-1RAP also plays an important role in promoting immune escape ability in cervical cancer by expressing CD47 on the surface of tumor cells.^19^ In HPV-negative Oropharyngeal Squamous Cell Carcinoma (OPSCC), it has been demonstrated that the IL-1/IL-1R axis produces chemokine CXCL8, leading to poor prognosis in OPSCC.^20^ IL-1RAP has also shown potential as a new therapeutic target in inflammatory diseases.^21^ Furthermore, blocking IL-1RAP inhibits pro-inflammatory cytokines (such as IFN) more effectively than blocking major receptors (IL-1R, ST2, IL-1Rrp2) alone. IL-1RAP also has therapeutic potential in tumors, and an ongoing treatment strategy is CAR-T cell therapy. Another strategy is to use Activated Antibody-mediated Cytotoxicity (ADCC) or antibody immunotherapy, which directly blocks IL-1RAP.^22^

IL-18RAP is a cell surface receptor that, when combined with IL-18, can trigger a series of signal transduction reactions. These reactions play a certain regulatory role in tumor cells’ growth, proliferation, and metastasis. In some tumors (lung, breast, colon, liver, stomach, kidney, bladder, ovarian, prostate, etc.), the expression level of IL-18RAP may change, thus affecting the development and progress of tumors.^23^ Notably, IL18RAP is differentially expressed in various cancers, and its levels are significantly downregulated in most cancers and correlated with clinical staging.^24^ Research has also demonstrated that IL-18RAP may promote tumor cell metastasis. In breast cancer, colorectal cancer, oral squamous cell carcinoma, and other tumors, the expression level of IL-18RAP is related to tumor metastasis and poor prognosis.^25^, ^26^ IL-18RAP may also be associated with tumor angiogenesis. Studies have shown that IL-18 can promote the formation of new blood vessels, which may contribute to the growth and spread of tumors.^27^ However, the expression of IL18RAP in head and neck tumors is still unclear, and its clinical significance and molecular biological role still need to be studied. In addition, IL-18RAP may be involved in the immune escape process of tumor cells. Tumor cells can inhibit the activity of NK and T-cells by expressing IL-18RAP, thereby evading immune system attacks. Further studies have also determined the distribution of IL18RAP in various cell types. We found that IL18RAP is expressed in various immune cells, including NK cells, CD8+ T-cells, and CD8+ Tex cells. Our study also confirmed the correlation between IL18RAP and CD8+ T-cells and its positive effect on prognosis, which is consistent with the descriptions of IL18RAP function by many scholars.^28^, ^29^, ^30^

SIGIRR (TIR8) is an interleukin-1 receptor-associated protein and a member of the TIR superfamily. Its function is to negatively regulate the innate immune response mediated by ILRs and TLR receptors.^31^ Additionally, SIGIRR is essential in regulating inflammatory responses induced by infectious diseases, tumors, and autoimmune diseases. Notably, the role of SIGIRR in tumor immune regulation is to suppress inflammation and immune cell activation by negatively regulating the inflammatory signaling pathway, thereby affecting the development and progression of tumors. SIGIRR can also negatively regulate the inflammatory signaling pathway of IL-1R/TLRs and inhibit inflammatory responses in the occurrence and development of different inflammatory-related diseases. Similarly, it has a certain inhibitory effect on tumor cells’ growth, proliferation, and metastasis. In colon cancer, methylation of SIGIRR can reduce its expression level in cancer tissue and may lead to enhanced proliferation, migration, and invasion ability of cancer cells.^32^ Furthermore, SIGIRR is considered to have the role of tumor suppressor genes in cervical, pancreatic, and ovarian cancer.^33^ At present, further research and exploration are needed on the specific mechanism and clinical significance of the role of SIGIRR in head and neck tumors.

## Conclusion

The importance of inflammation in the occurrence and development of tumors has been fully confirmed. IL-1 is an attractive target choice for tumor drug development and a potential molecular target for improving the prognosis of cancer patients. This article explores the targets that affect local CD8+ T-cell infiltration in tumors and subsequently affect patient prognosis (IL-1A, IL1RAP, IL18RAP, SIGIRR), which may also become a breakthrough in improving tumor treatment efficacy.

## Funding

National Natural Science Foundation of China (82071032 and 82072997).

## Conflicts of interest

The authors declare no have conflicts of interest.
